# Supplementary material for: Integrins mediate placental extracellular vesicle trafficking to lung and liver in vivo
Source: Sci Rep. 2021 Feb 18;11:4217. doi: 10.1038/s41598-021-82752-w (PMC7893009; doi:10.1038/s41598-021-82752-w)
Supplement: Supplementary file 1 — Supplementary Information [file 41598_2021_82752_MOESM1_ESM.pdf]

Supplementary Information

INTEGRINS MEDIATE PLACENTAL EXTRACELLULAR VESICLE TRAFFICKING TO LUNG AND LIVER IN VIVO

Sean L. Nguyen, Soo Hyun Ahn, Jacob W. Greenberg, Benjamin W. Collaer, Dalen W. Agnew, Ripla Arora, and Margaret G. Petroff

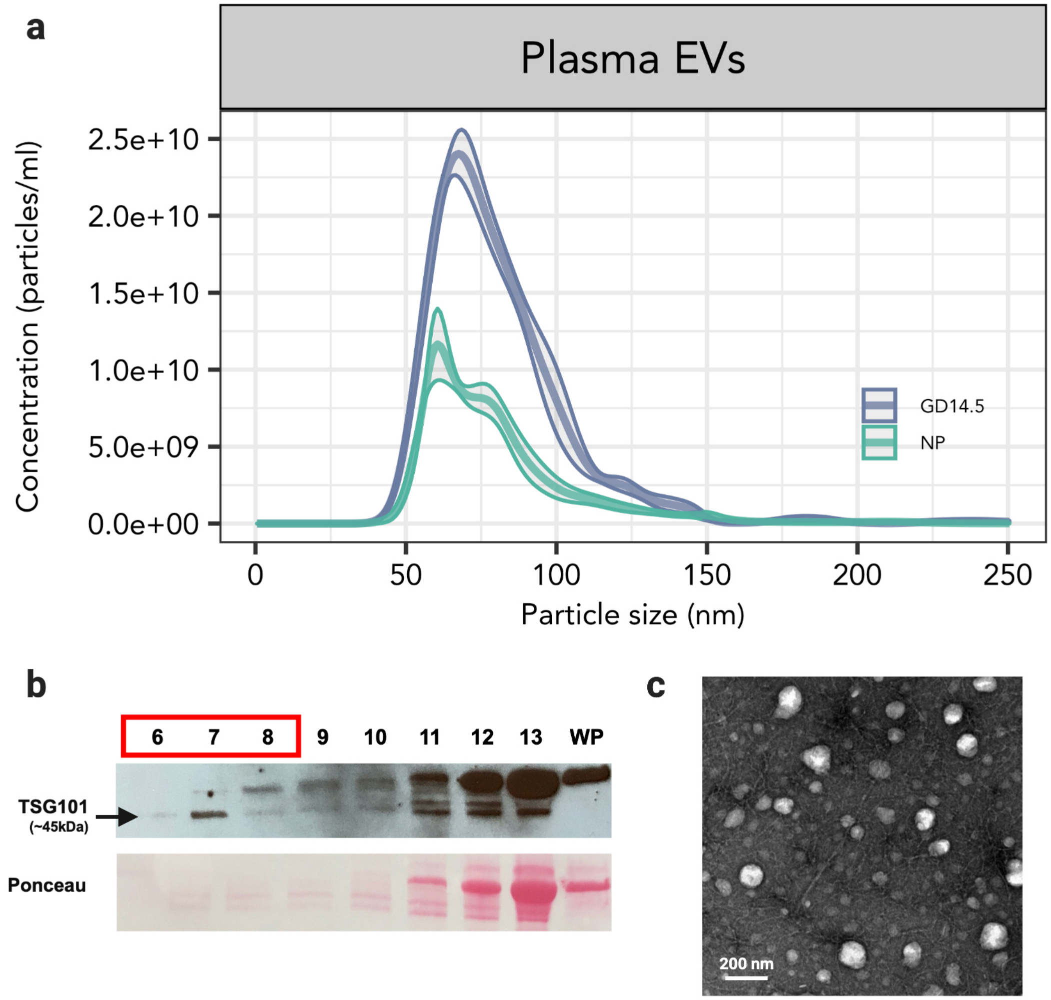

Supplemental Figure 1. Isolation and validation of plasma EVs.

A. Representative concentration and size distribution analysis of plasma EVs from non-pregnant (NP) and gestational day (GD) 14.5 mice obtained by ultracentrifugation and analyzed by NanoSight. The thick middle line in each distribution curve represents mean concentration, and the shaded area bound by narrower lines represents SEM. Data represent five technical replicates of three different animals in each group.
B. Western blot analysis of plasma EVs from a GD14.5 mouse. Lane numbers represent size exclusion chromatography fractions; red box indicates fractions used for in vivo experiments. WP, whole plasma. Representative data from three independent experiments.
C. Transmission electron microscopy of plasma EVs (fractions 6-8) from a nonpregnant mouse (representative image of n = 3).

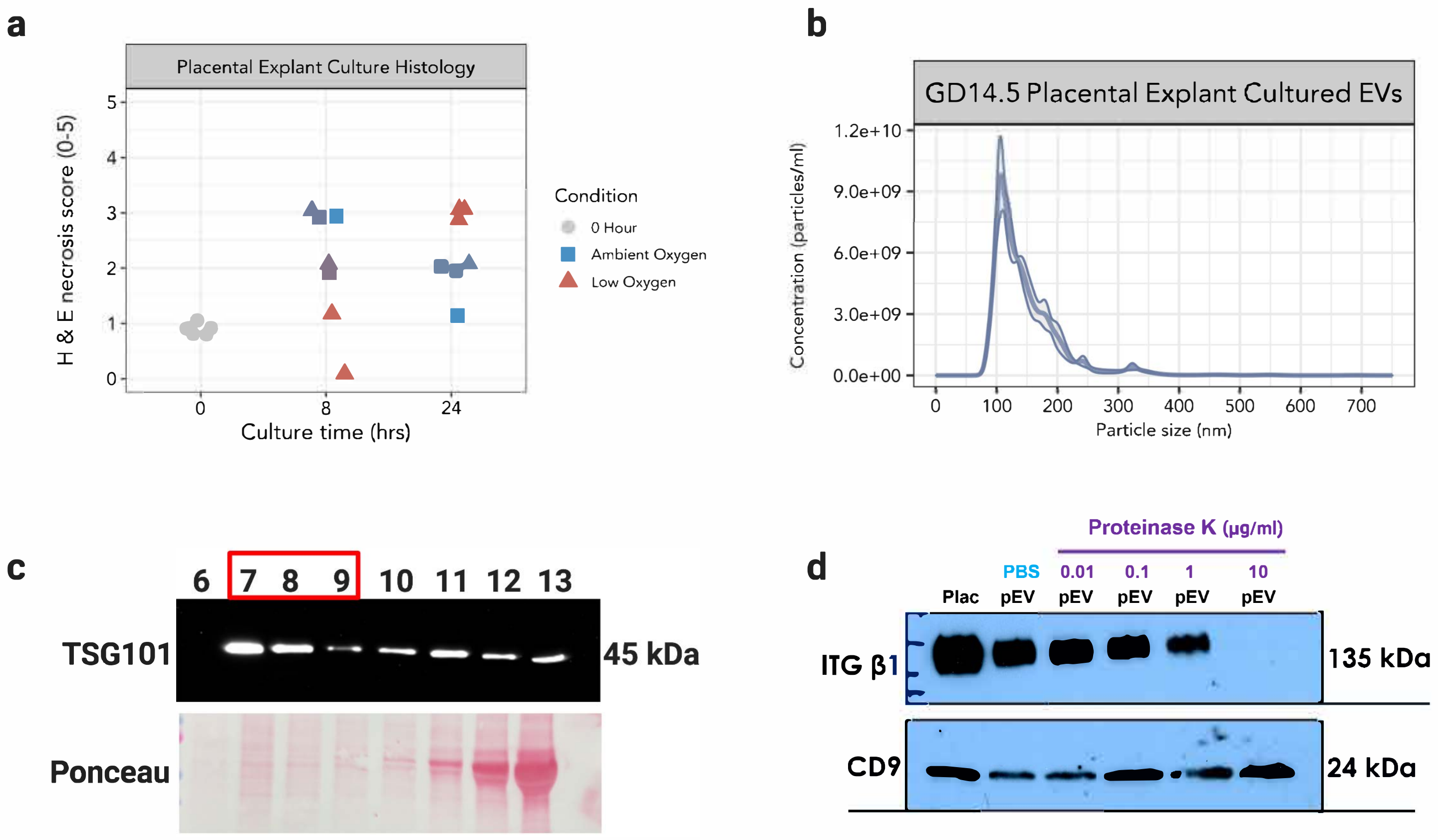

Supplemental Figure 2. Validation of placental explant culture.

A. GD14.5 placentas were cultured under ambient or low (8% oxygen) conditions for 8 or 24 hours and processed for histological analysis. Uncultured placentas were dissected and processed immediately after dissection (0 hr). Hematoxylin and eosin-stained sections were subjected to blinded scoring for degree of necrosis on a scale of 0-5.
B. Representative histogram of placental EVs analyzed by nanoparticle tracking analysis.
C. Western blot analysis of placental EVs from culture supernatants. Numbers over lanes represent fractions from size exclusion chromatography; red box indicates fractions used for in vivo experiments.
D. Western blot of placenta (plac) and placental EVs treated with PBS (negative control) or increasing concentrations of proteinase K. ITGβ1, which was strongly expressed by placental EVs, was efficiently removed by 10 µg/ml proteinase K, which did not remove immunoreactivity for the exosomal marker CD9. Blot is representative of three independent experiments.

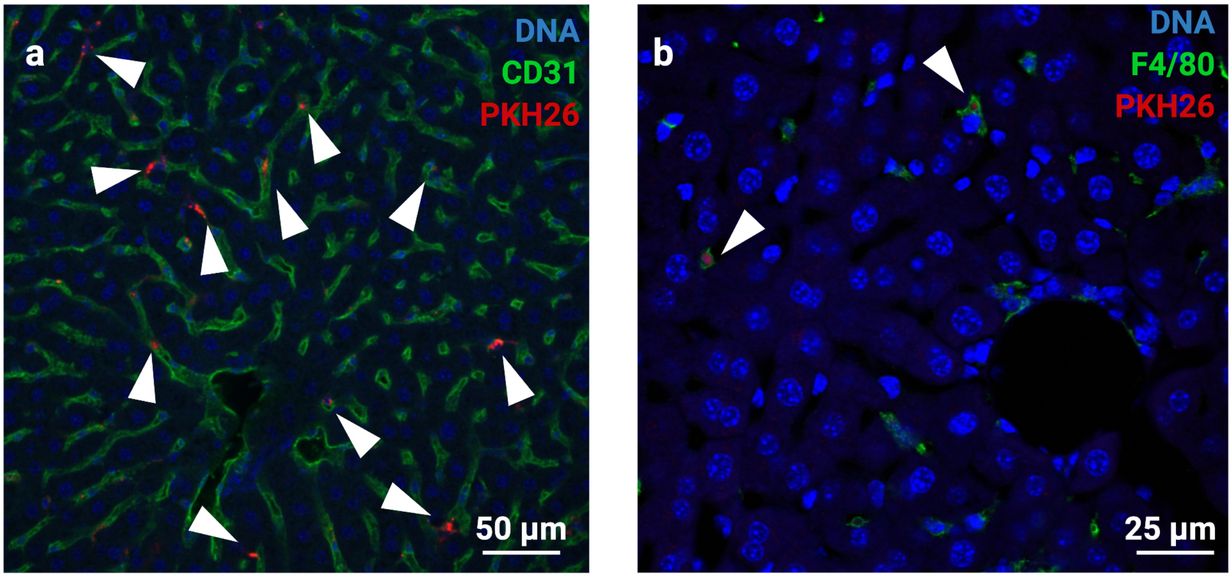

Supplemental Figure 3. Placental EV Localization in Liver.

A. Immunofluorescence confocal microscopy showing colocalization of placental EV (red punctate fluorescence) with CD31+ endothelial cells.

B. Immunofluorescence colocalization of placental EV with F4/80+ macrophages. Arrowheads highlight colocalization. Representative images from three independent experiments.

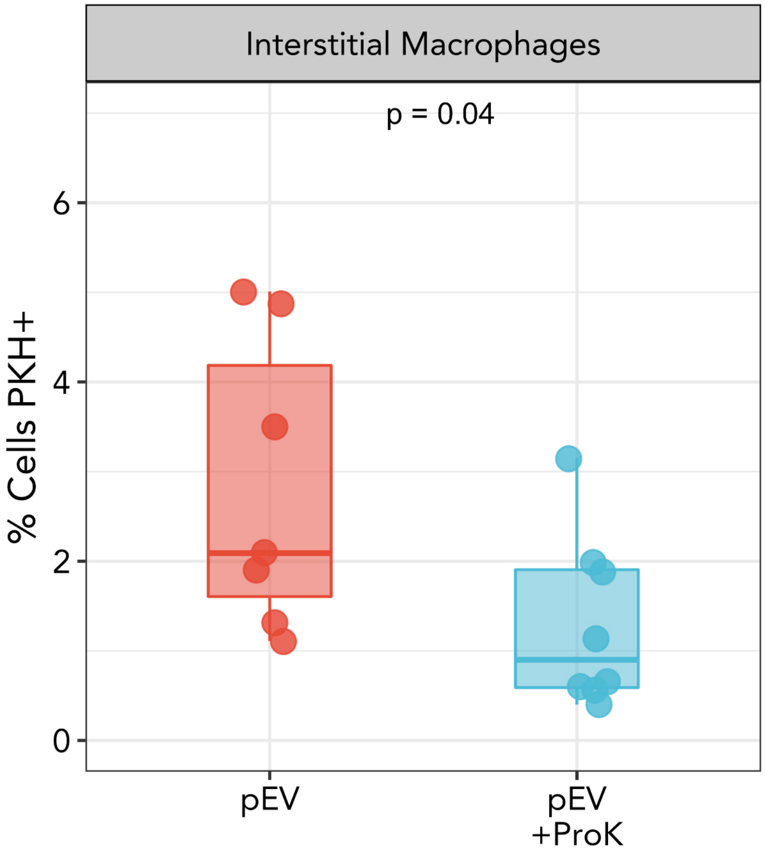

Supplemental Figure 4. Proteinase K inhibits pEV localization to lung interstitial macrophages.
Flow cytometric quantification of placental EV localization to interstitial macrophages in lung. Points represent individual biological replicates; data were analyzed by Welch's T-test.

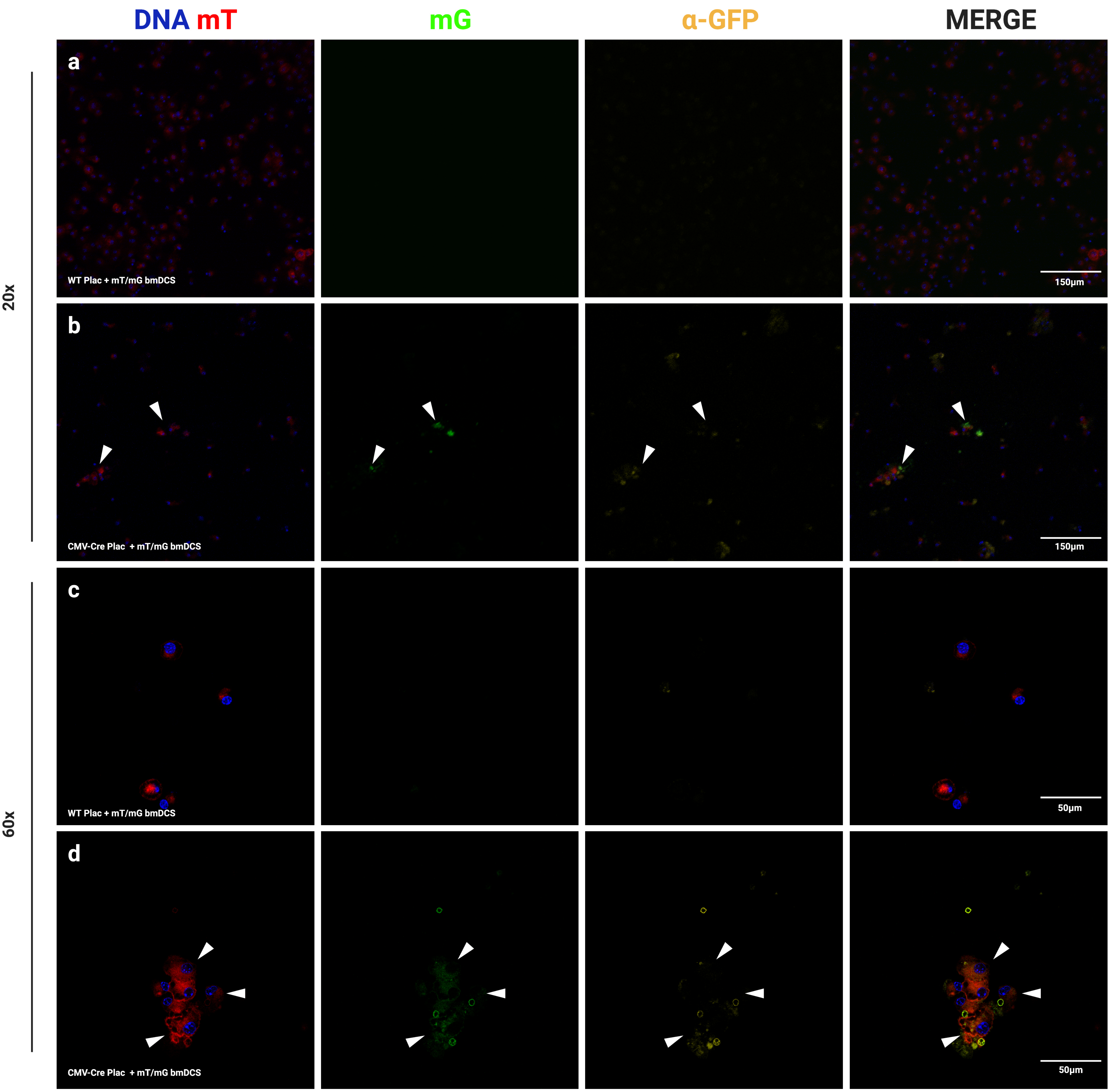

Supplemental Figure 5. In vitro recombination of BMDC co-cultured in the presence of CMV-Cre placentas.
Reporter BMDC from mT/mG mice were co-cultured with WT or CMV placentas and viewed by confocal microscopy for absence (mT) or presence (mG) of recombination. Results of mG were confirmed using an AF-647-conjugated anti-GFP antibody (α-GFP). Representative confocal microscopy of reporter BMDCs co-cultured with WT (left) or CMV-Cre (right) placentas. Arrowheads point to GFP foci in cells.
A, C: BMDC cocultured with WT placentas.
B, D: BMDC cocultured with CMV-Cre placentas.

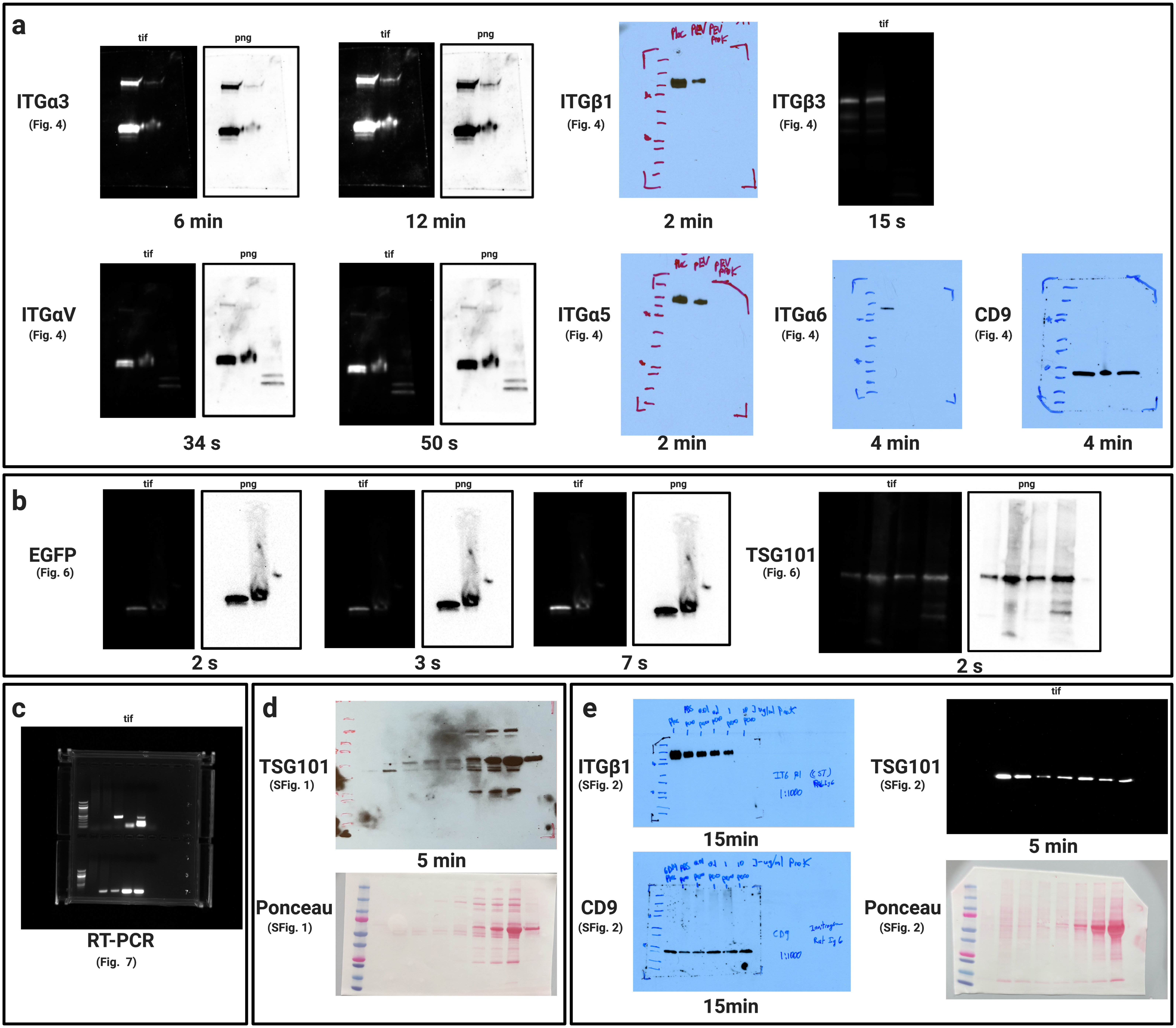

Supplemental Figure 6. Original Western blot and gel images.
Representative full length western blot images for relevant figures, including multiple exposures with raw TIF images and corresponding unmanipulated PNG images whre possible.

a. Figure 4.
b. Figure 6.
c. Figure 7.
d. Supplemental Figure 1.
e. Supplemental Figure 2.
